# Catheter-induced Multiple Non-proximal Coronary Spasm in a Patient Presenting with Myocardial Infarction

**DOI:** 10.7759/cureus.7456

**Published:** 2020-03-29

**Authors:** Kerim Esenboga, Emir Baskovski, Nil Ozyuncu, Eralp Tutar

**Affiliations:** 1 Cardiology, Ankara University School of Medicine, Ankara, TUR

**Keywords:** coronary spasm, myocardial infarction, case report, interventional cardiology

## Abstract

Interventional cardiologists encounter a wide range of lesions that cannot be angiographically distinguished from fixed atherosclerotic obstructive disease. In this case report, we document vasospasm at multiple sites in the coronary territory in a patient presenting with acute coronary syndrome. A 61-year-old woman was referred to our hospital with typical chest pain lasting approximately 1 h. After performing the left coronary artery angiography, a severe tubular stenosis was detected in circumflex (Cx) artery. Diffuse spasm was observed in the right coronary artery (RCA) and it resolved after intracoronary administration of nitroglycerin. After performing left system angiography again, severe stenosis in Cx artery was also completely resolved. Our finding is of clinical importance in that it is more likely to simulate a constant coronary stenosis than would have spasm occurred proximally. The clinical importance of our report is that a catheter-induced vasospasm (CIV) may simulate fixed coronary stenosis, not always osteally and in some instances at multiple sites. Awareness of this phenomenon and liberal use of nitroglycerin in any patient with discrete luminal narrowing, even when an ostial “lesion” is not present, can help to avoid misinterpreting CIV as an atherosclerotic lesion.

## Introduction

Interventional cardiologists encounter a wide range of lesions that cannot be angiographically distinguished from atherosclerotic obstructive disease. While there are many etiologic factors that lead to coronary vasospasm, catheter-induced vasospasm (CIV) arises during diagnostic coronary angiography or percutaneous coronary intervention (PCI). The incidence varies, although in one large observational, retrospective study CIV in right coronary artery (RCA) was found to be 0.75% [1]. Some reports suggest that CIV may be associated with increased propensity to develop spontaneous clinical episodes of coronary spasm [2-6].

Herein, we report a patient presenting with acute coronary syndrome who on coronary angiography had vasospasm at multiple sites across the coronary territory.

## Case presentation

**Figure 1:**
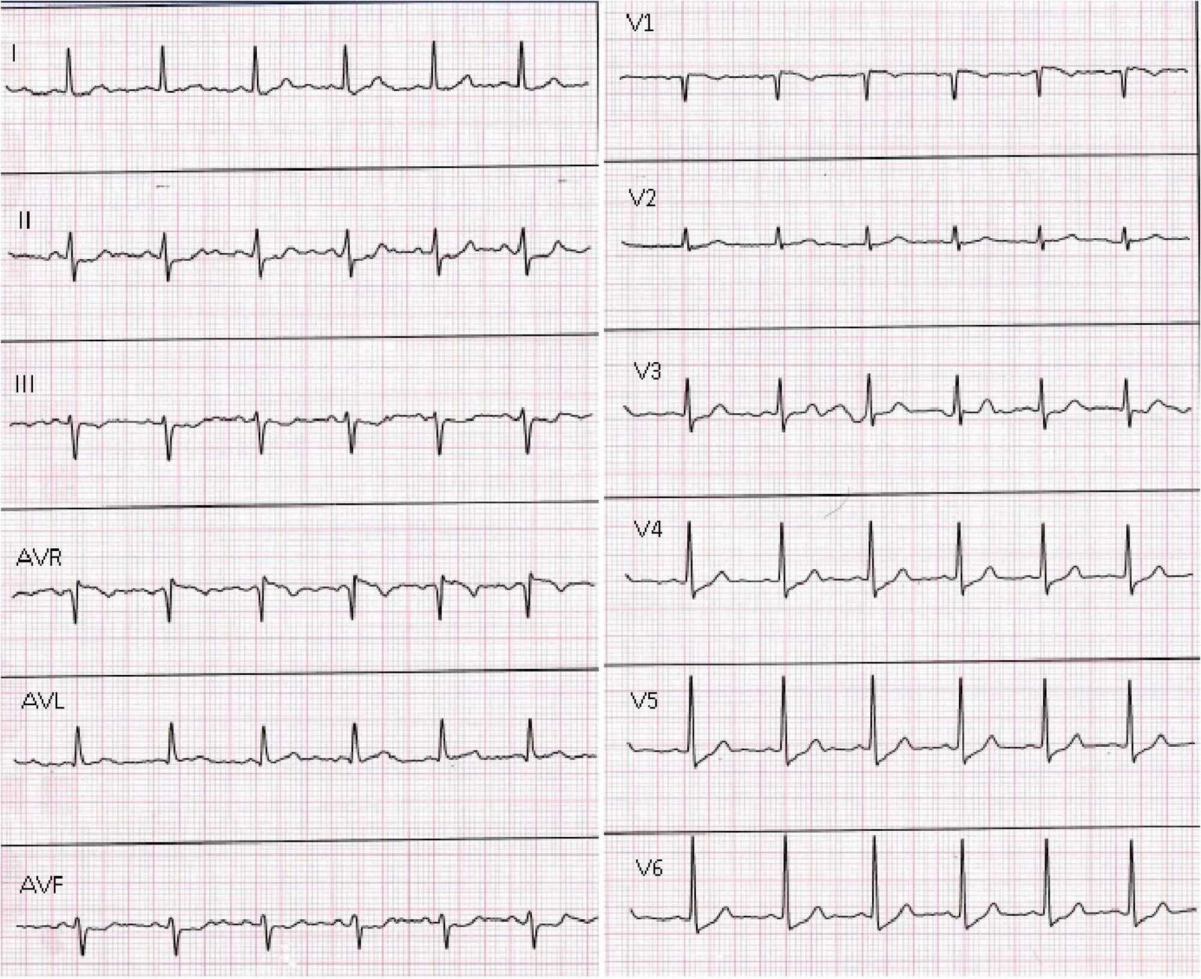
Electrocardiography at the time of admission.

A 61-year-old woman presented to a local hospital with typical chest pain lasting for approximately 1 h. The patient history was significant for coronary angiography performed three years prior to this presentation due to typical angina pectoris induced by exercise, revealing normal coronary arteries. On this presentation, serum troponin level was high and her electrocardiogram revealed 0,5-1 mm ST segment depression in precordial derivations (Figure 1). The patient was referred to our hospital for invasive management of myocardial infarction.

**Figure 2:**
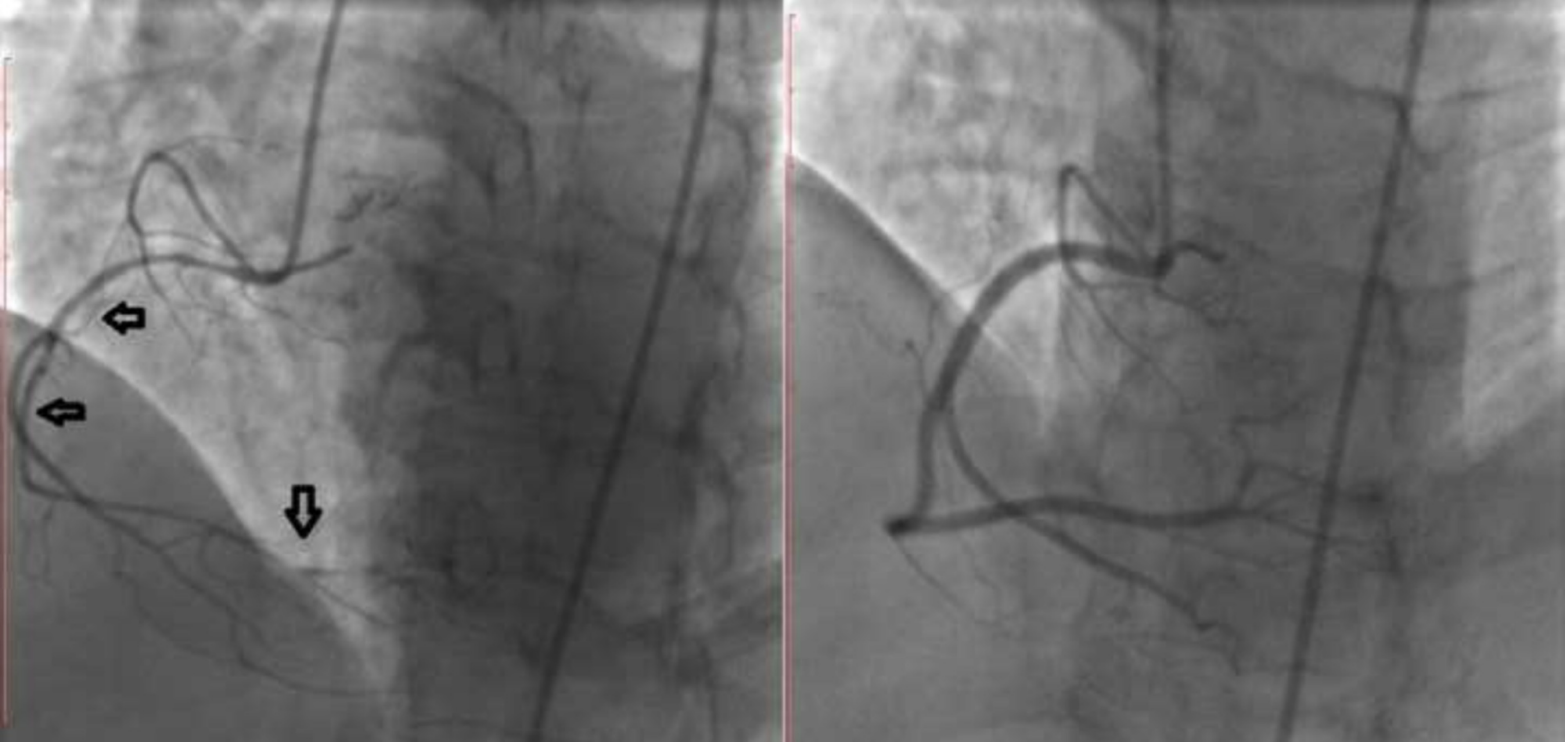
Right coronary angiography. Diffuse spasm in the right coronary artery (left) completely resolved after intracoronary administration of nitroglycerin (right).

**Figure 3:**
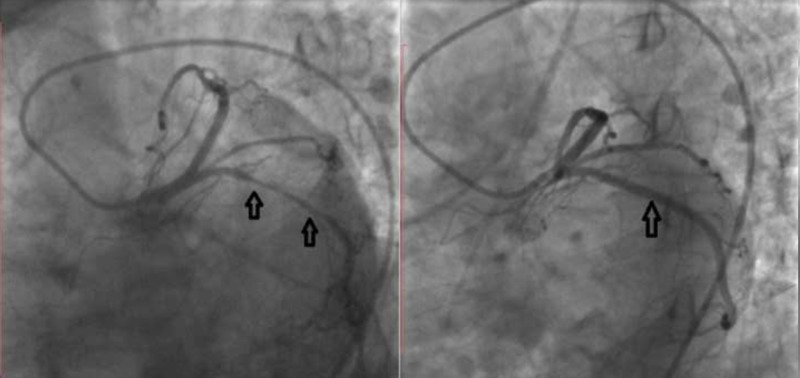
Left coronary angiography. Severe stenosis in circumflex artery (left) resolved completely after administration of nitroglycerin (right).

Except for hypertension treated with irbesartan/hydrochlorothiazide and metoprolol, the patient had no other risk factors for coronary artery disease (CAD). She denied substance abuse, including cocaine abuse. No structural heart disease was detected by transthoracic echocardiography. Early invasive management was planned, therefore coronary angiography was performed. Left coronary angiography, performed with a 6 French Judkins diagnostic catheter, revealed a severe tubular stenosis in circumflex (Cx) artery, evident on various left and right anterior oblique projections. No other lesions (including atherosclerotic plaques) were observed in the left coronary system. On right coronary angiography, performed with a 6F right Judkins diagnostic catheter, diffuse spasm of RCA was observed, most prominent in the distal vessel. A second right coronary angiography, performed 30 s following a 200 peg nitroglycerin intracoronary bolus, revealed complete resolution of spasm (Figure 2). Having suspected spasm as etiology of the severe lesion in Cx, angiography was repeated, revealing complete resolution (Figure 3). The patient did not report any symptoms throughout the procedure. Resolution of the RCA and Cx stenosis with nitroglycerin suggested CIV in both territories. Furthermore, concentric lesions with otherwise angiographically normal vessels along with morphology of both stenoses were more compatible with arterial vasospasm rather than an atherosclerotic plaque. Beta blocker therapy was discontinued and the patient was discharged on oral diltiazem. On follow-up, she remained symptom-free with medical treatment.

## Discussion

Coronary artery spasm is one of the etiopathogenetic mechanisms of acute coronary syndromes in patients with no other pathology in coronary arteries [7-9]. Patient may even undergo coronary artery bypass surgery due to a misdiagnosis in which coronary artery spasm was misinterpreted as fixed atherosclerotic obstruction [10].

In one report, catheter-induced spasm was observed in 8 of 63 patients (13%) who have Prinzmetal’s angina [3]. Raizner et al. and Matsuda et al. have also described patients with both spontaneous and CIV observed during the same angiographic examination [4-5]. Sueda et al. have reported that pharmacologic spasm provocation was positive in 88.9% patients who have CIV [1]. The strong association between spontaneous coronary artery spasm and CIV suggests that the most probable etiologic factor of acute coronary syndrome in this case is variant angina.

The incidence of CIV varies by physician expertise and usage of different types of catheters, although the later was not found to be statistically significant in one study [1]. Besides diagnostic procedures, the catheter-induced spasm is also frequently observed during percutaneous intervention to coronary artery bypass grafts or native coronary vessels [8]. Platelet accumulation at the catheter tip with secretion of vasoactive agents, activation of stress receptors, and mechanical stretch triggering a myogenic reflex are etiopathogenetic factors taking role in the development of CIV [11-14]. RCA is more likely affected by CIV, however, it has also been documented in coronary arteries in denervated patients following heart transplantation and left main coronary artery [15-16]. Although vasospasm usually resolves by catheter removal and nitroglycerin injection, persistent cases have also been reported [17]. In addition, although rare, vasospasm in nonproximal coronary segments in CIV has been reported [18]. The distinguishing feature of our case is bilateral occurrence of CIV. Documented bilateral CIV is very rare except in heart transplant patients [19].

To our knowledge, there are no trials evaluating long-term management of CIV, however, similar pathophyisology and high prevalence of CIV in variant angina and vice versa suggest that calcium channel blockers and oral nitrates may be a good candidate for therapy [20]. We have treated our patient with oral diltiazem, having in mind that vasospastic angina was probably the etiologic factor of ACS. Rare refractory cases may need stenting to prevent acute coronary spasm.

The clinical importance of our report is that a CIV may simulate fixed coronary stenosis, not always osteally and in some instances at multiple sites. Awareness of this phenomenon and liberal use of nitroglycerin in any patient with discrete luminal narrowing, even when an ostial “lesion” is not present, can help to avoid misinterpreting CIV as an atherosclerotic lesion.

## Conclusions

In conclusion, it should be kept in mind that CIV can occur in multiple sites, as in our case, away from the catheter tip. Adequate doses of intracoronary nitroglycerin should be administered prior to coronary angiography procedure whenever uncertainty regarding diagnosis exists.
